# Methyl prednisolone vs Dexamethasone in Management of COPD Exacerbation; a Randomized Clinical Trial

**Published:** 2017-01-11

**Authors:** Mohammad Emami Ardestani, Elham Kalantary, Vajihe Samaiy, Keramat Taherian

**Affiliations:** 1 Department of Internal Medicine, Isfahan University of Medical Sciences, Isfahan, Iran.; 2 Department of Emergency Medicine, Isfahan University of Medical Sciences, Isfahan, Iran.

**Keywords:** Methylprednisolone, dexamethasone, pulmonary disease, chronic obstructive

## Abstract

**Introduction:**

Corticosteroids are routinely used in management of chronic obstructive pulmonary disease (COPD) exacerbation. The main purpose of present study was to compare the efficacy of methyl prednisolone (MP) and dexamethasone (DXM) for this purpose.

**Methods:**

Adult COPD patients entered the present clinical trial. All patients received standard treatment on admission and were then divided into 2 groups of intravenous MP and DXM. Patients were asked to rate their shortness of breath; sputum volume and viscosity; dyspnea; cough; and general wellbeing on a 0-5 scale. Baseline parameters such as O_2_ saturation, arterial blood gas parameters, and white blood cell (WBC) count were compared on admission and day 7 and 14 of therapy using SPSS 22.

**Results:**

68 patients were randomly allocated to 2 groups of 34 (82.4% male). The baseline characteristics of the two groups were similar (p < 0.05). Comparison of treatment outcomes for the 7^th^ day showed a significant difference between the 2 groups only regarding cough (p = 0.047), HCO3 (p < 0.001), and O_2_ saturation (p = 0.042). On day 14 the 2 groups were different only regarding cough (p = 0.048) and sputum viscosity (p = 0.011). There was a significant difference between the two groups regarding trend of changes in dyspnea (p = 0.02; DXM >> MP) and cough (p = 0.035; MP >> DXM). There were no significant differences between the two medications regarding side effects on 7^th^ and 14^th^ day after treatment.

**Conclusion:**

It seems that MP and DXM have similar efficacy and side effects in treatment of COPD exacerbation and selecting drug of choice would better be based on the most prominent symptoms of patients on admission.

## Introduction

Chronic obstructive pulmonary disease (COPD) is the most prominent cause of morbidity and mortality in developed countries and has raised to become the third cause of mortality worldwide (1-4).

In accordance with international guidelines (GOLD), the diagnosis of COPD should be confirmed with a spirometry result showing a post-bronchodilator value of FEV1/FVC ratio < 0.7 (5).

The key interventions on COPD exacerbation is to control airway inflammation, relieve airflow obstruction and improve ventilation (6). Corticosteroids are a large group of drugs used in COPD exacerbation and are chosen based on clinical presentations of the patient. It has been shown that using systemic corticosteroids for treating exacerbation of COPD leads to reduced failed treatments and improves lung function in the first 72 hours and shortens hospital stay in non-critically ill patients (7-10). Different types of corticosteroids with different characteristics, such as methyl prednisolone (MP) and dexamethasone (DXM), have been used in this regard (11, 12). 

Based on the above-mentioned points, the main purpose of present study was to compare the efficacy of MP and DXM in treatment of COPD exacerbation. 

## Methods

***Study design and setting***

This is a prospective, randomized, single-blind trial conducted between 2013 and 2014 in Al-Zahra Hospital, Isfahan, Iran. The institutional review board of Isfahan University of Medical Sciences approved the study protocol. All patients gave their written informed consent and the protocol was approved by the hospital’s Ethics Committee. The researchers adhered to the principles of Helsinki Declaration over the course of the study. The protocol of the present study was registered on Iranian registry of clinical trials under IRCT number:

***Participants***

All studied patients met the criteria outlined in the guidelines for diagnosis and management of COPD, established by the GOLD standard (13). Adult COPD patients with acute respiratory distress, increased cough frequency and severity, increased sputum volume, and/or increased wheezing for 24 hours or more were eligible for entry to the study. Patients with history of asthma or atopy, onset of respiratory distress before the age of 35 years, absence of spirometric data, or having received oral or intravenous steroids in the month prior to presentation were excluded. The 68 cases were randomly allocated to two equal groups of 34, using simple randomization method. Patients and data analyser were blinded to type of treatment. 

***Intervention***

On admission to ED, oxygen therapy was performed until O_2_ saturation raised to above 88-90%. In addition, all individuals in both groups received a combination of a macrolide (azithromycin) and a third generation cephalosporin (ceftriaxone); nebulized β_2_-agonist (salbutamol), anticholinergic agent (ipratropium bromide), and inhaled corticosteroid (budesonide). They were then divided into 2 groups of MP and DXM for receiving intravenous (IV) corticosteroid.

The MP group received 2 mg/kg/day MP intravenously for 3 days. Then the dose was reduced to 40 mg for 3 days and switched to 30 mg/day of oral prednisone, which was tapered every 3 days with 5 mg decrease in dosage. Then inhaled budesonide, 400 micrograms, twice a day was prescribed. Prednisone was tapered for 2 weeks, and then ceased. Inhaled corticosteroid had to be used for at least 3 months continuously. 

The second group (DXM group) received 0.375 mg/kg DXM per day, and then its dosage was gradually tapered. After 7 to 14 days, the drug was replaced by 30 mg/day methyl prednisone, and continued by the same protocol as MP group.

***Assessments***

Patients were assessed within an hour of admission and then every day for two weeks to evaluate the therapeutic effect of treatment and the side effects.

Questions were asked about shortness of breath; sputum volume and viscosity; dyspnea; cough; and general wellbeing on admission and on every day of therapy. Patients were asked to score each symptom from 0 (much better than usual) to 5 (much worse than usual).

Baseline parameters such as O_2_ saturation, arterial blood gas parameters, and white blood cell (WBC) count were evaluated on admission and on day 7 and 14 of therapy.

Potential corticosteroid side effects such as mood changes, heartburn, overt gastrointestinal bleeding, and blood sugar disturbance were recorded in every visit. 

***Statistical analysis***

Sample size was calculated to be 33 in each group based on Zα = 1.96, Zβ = 0.84, s = 12.4, and d = 8.6. All data from the patients were analysed using independent sample T-test or Mann-Whitney U test for quantitative variables and Chi-square test for qualitative variables. Chi-square for trend analysis was used to compare trend of changes in sputum volume and viscosity as well as dyspnea and cough between the two groups. A value of p < 0.05 was considered to indicate statistical significance. All data are reported as mean ± standard deviation (SD). Analyses were done using SPSS software version 22.0 (SPSS Inc., Chicago, IL).

## Results

**Figure 1:**
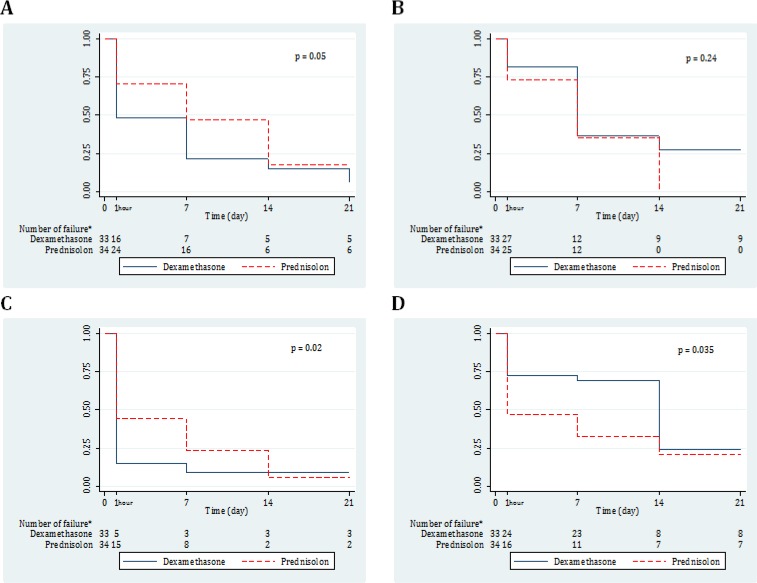
Failure rate of dexamethasone and prednisolone in treatment of sputum volume (A), sputum viscosity (B), dyspnea (C), and cough (D).

68 COPD patients were randomly allocated to 2 equal groups of DXM (82.3% male) and MP (82.4% male). The mean age of DXM and MP groups were 74.67 ± 1.79 and 73.35 ± 2.25 years, respectively (p = 0.648). Baseline characteristics of the two groups were compared in table 1. There were no significant differences between the two medications regarding side effects on 7^th^ and 14^th^ day after treatment (table 2). Outcome of studied groups on 7^th^ and 14^th^ days after treatment were presented in table 3 and figure 1. 

*7-day treatment outcome*

Comparison of treatment outcomes for the 7th day showed a significant difference between the 2 groups only regarding cough (p = 0.047; remission: MP: 23 (67.7%) vs. DXM: 16 (48.5%)), HCO3 (MP: 22.58 ± 3.62 vs DXM: 25.66 ± 2.82; p < 0.001), and O2 saturation (MP: 87.71 ± 3.15 vs DXM: 89.24 ± 2.88; p = 0.042).

*14-day treatment outcome*

Comparison of treatment outcomes for the 14th day showed a significant difference between the 2 groups only regarding cough (p = 0.048; remission: MP: 27 (79.4%) vs. DXM: 25 (75.7%)) and sputum viscosity (p = 0.011; remission: MP: 34 (100%) vs. DXM: 24 (72.7%)).

*Trend of changes*

There were not any significant differences between the 2 groups regarding trend of changes in sputum volume (p = 0.05), sputum viscosity (p = 0.24), O_2_ saturation (p = 0.87), PaCO_2_ (p = 0.83), HCO_3_ (p = 0.12), serum pH (p = 0.42), WBC count (p = 0.24), on 7^th^ and 14^th^ day after treatment. There was a significant difference between the two groups regarding trend of changes in dyspnea (p = 0.02; DXM >> MP) and cough (p = 0.035; MP >> DXM).

## Discussion

The findings of this study demonstrated that although both treatments are effective and similar in most treatment characteristics, MP is better for reducing cough, and sputum viscosity, while DXM showed a significantly better trend regarding dyspnea treatment and increased O_2_ saturation, more significantly on the 7^th^ day. Although a significant difference was detected between the 2 groups regarding HCO_3_, it is not clinically significant. 

**Table 1:** Baseline characteristics of the patients in the two groups

**Variables **	**Dexamethasone**	**Methylprednisolone**	**p**
**Duration of disease**			
Years	8.02 ± 5.25	8.64 ± 4.61	0.608
**Shortness of breath**			
None	4 (11.8)	0 (0)	0.792
Mild	3 (8.8)	6 (17.6)	
Moderate	7 (20.6)	8 (23.5)	
Severe	20 (58.8)	20 (58.8)	
**Cough **			
None	4 (11.8)	0 (0)	0.556
Mild	2 (5.9)	3 (8.8)	
Moderate	9 (26.5)	11 (32.4)	
Severe	19 (55.9)	20 (58.8)	
**Sputum volume **			
None	8 (23.5)	0 (0)	0.081
Mild	5 (14.7)	8 (23.5)	
Moderate	5 (14.7)	5 (14.7)	
Severe	16 (47.1)	21 (61.8)	
**High sputum viscosity**			
Yes	18 (52.9)	21 (61.8)	0.624
No	16 (47.1)	13 (38.2)	
**O**_2_ ** saturation **			
On arrival	79.26 ± 6.61	78.65 ± 8.24	0.734
**PaCO**_2_			
On arrival	64.85 ± 10.00	64.85 ± 12.52	1.000
**HCO**_3_			
On arrival	26.55 ± 5.37	27.76 ± 5.52	0.365
**pH**			
On arrival	7.32 ± 0.04	7.30 ± 0.05	0.218
**White blood cell count/mm** ^3^			
Before treatment	8.49 ± 3.6	7.35 ± 3.08	0.164

**Table 2:** Comparison of drug side effects between the two groups on 7th and 14th days

**Complications/side effects**	**Number (%)**		**P**
	**Dexamethasone**	**Methylprednisolone**	
**Gastrointestinal bleeding**			
7	3 (9.1)	2 (5.9)	0.67
14	3 (9.1)	5 (14.7)	0.71
**Mood change**			
7	0 (0)	2 (5.9)	0.49
14	0 (0)	3 (8.8)	0.24
**Heart burn**			
7	6 (18.2)	4 (11.8)	0.51
14	9 (27.3)	6 (17.6)	0.39
**Blood sugar disturbance**			
7	6 (18.2)	4 (11.8)	0.51
14	9 (27.3)	6 (17.6)	0.39

**Table 3:** Treatment outcome of dexamethasone (DXM) and methylprednisolone (MP) groups

**Variables**	**Outcome (day)**			**p**
	**Baseline**	**7** ^th^	**14** ^th^	
**Improvement in sputum volume**				
DXM	17 (51.51)	26 (78.79)	28 (84.85)	0.05
MP	10 (29.41)	18 (52.94)	28 (82.35)	
**Improvement in sputum viscosity**				
DXM	6 (18.18)	21 (63.64)	24 (72.72)	0.24
MP	9 (26.47)	22 (64.70)	34 (100.0)	
**Improvement in dyspnea**				
DXM	28 (84.85)	30 (90.91)	30 (90.91)	0.02
MP	19 (55.88)	26 (76.47)	32 (94.12)	
**Improvement in cough**				
DXM	9 (27.27)	10 (30.30)	25 (75.76)	0.035
MP	18 (52.94)	23 (67.65)	27 (79.41)	
**O**_2_ ** Saturation**				
DXM	79.26 ± 6.61	87.70 ± 3.19	90.97 ± 3.31	0.87
MP	78.64 ± 8.24	89.23 ± 2.89	92.52 ± 2.74	
**PaCO**_2_				
DXM	64.85 ± 10.00	49.50 ± 6.67	44.32 ± 3.35	0.83
MP	64.85 ± 12.52	50.32 ± 4.46	45.32 ± 3.91	
**HCO**_3_				
DXM	26.55 ± 5.37	22.58 ± 3.63	23.12 ± 2.17	0.12
MP	27.76 ± 5.52	25.73 ± 2.75	23.91 ± 2.23	
**Serum pH**				
DXM	7.32 ± 0.04	7.32 ± 0.04	7.36 ± 0.05	0.42
MP	7.30 ± 0.04	7.32 ± 0.04	7.36 ± 0.03	
**White blood cell count/mm** ^3^				
DXM	8.49 ± 3.6	9.16 ± 6.25	6.81 ± 1.92	0.24
MP	7.35 ± 3.08	11.89 ± 14.29	6.80 ± 1.95	

Despite accepted effects of corticosteroids on airflow obstruction relief and being used since 1950, drug of choice, optimal dose, and duration of treatment remain unclear (14). Several studies have demonstrated that corticosteroids can significantly improve patients’ symptoms and lung function (15-18).

Li et al. had compared MP with DXM, with the same dosage as this study, in acute exacerbation of COPD and believed that DXM was less effective than MP, as cough and sputum in MP group were relieved more quickly than DXM group (6). The present study also showed a significant difference in cough relief in MP group. Yet, although the change in trend was similar in both groups, MP group showed significantly better sputum viscosity remission only on the 14^th^ day. 

Zhao et al. also compared these drugs in severe pediatric asthmatic bronchitis and concluded that MP is superior to DXM for this purpose. However, in contrast to the present findings, they demonstrated that MP retrieves hypoxia quickly (19). 

Li et al. also demonstrated that, there was no striking difference in blood gas improvement between the two groups, although the effect of MP seemed slightly superior to that of DXM (6). This is in contrast to our results showing higher O_2_ saturation on the 7^th^ day in DXM group. 

Andre et al. compared MP and DXM in premature infants who were at risk of chronic lung diseases and concluded that MP is as efficient as DXM with fewer side effects (20). In this study, we found that they were both effective, but they were not significantly different regarding side effects. 

DXM has well-known pharmacologic properties, including duration of action of up to 72 hours, a relatively long half-life, and excellent bioavailability (21). As a result, it has been proffered as an alternative to prednisone that may allow shorter treatment regimens and improved compliance. Joel Kravitz et al. indicated that 2 days of oral DXM is at least as effective as 5 days of prednisone in the treatment of mild to moderate asthma exacerbation. Relapse and treatment failure rates were equivalent in both groups (22). 

It seems that selection of corticosteroids in COPD exacerbation should be performed based on the most prominent symptom of patients. In cough prominent cases, MP seems to have better effects and in sputum prominent cases, DXM is superior. 

## Limitations:

Single blinding (drug administrator was not binded), lack of long term follow-up regarding times of readmission with exacerbation, and qualitative measurement based on patient declaration are among the most important limitations of present study.

## Conclusion:

Our study showed that MP and DXM have similar efficacy and side effects in treatment of patients with COPD exacerbation and selecting drug of choice would better be based on the most prominent symptoms of patients.
